# Impact of Micronized Salt in Reducing the Sodium Content in Fresh Sausages

**DOI:** 10.3390/foods13030459

**Published:** 2024-02-01

**Authors:** Chimenes D. L. de Araújo, Monique M. Krauskopf, João A. S. Manzi, José A. L. Barbosa, Cecylyana L. Cavalcante, Gabriela Feltre, Juan D. Rios-Mera, Erick Saldaña, Carmen J. Contreras Castillo

**Affiliations:** 1 Departamento de Agroindústria, Alimentos e Nutrição (LAN), Escola Superior de Agricultura “Luiz de Queiroz” (ESALQ), Universidade de São Paulo (USP), Piracicaba 13418-900, SP, Brazil; chimenesdarlan@usp.br (C.D.L.d.A.); mm.krauskopf@usp.br (M.M.K.); joao_manzi@usp.br (J.A.S.M.); cecylyanaleite@usp.br (C.L.C.); gabriela.feltre@usp.br (G.F.); 2 Departamento de Zootecnia, Escola Superior de Agricultura “Luiz de Queiroz” (ESALQ), Universidade de São Paulo (USP), Piracicaba 13418-900, SP, Brazil; andrew.lira@usp.br; 3 Instituto de Investigación de Ciencia y Tecnología de Alimentos (ICTA), Universidad Nacional de Jaén, Jaén 06800, Peru; juan.rios@unj.edu.pe; 4 Sensory Analysis and Consumer Study Group, Escuela Profesional de Ingeniería Agroindustrial, Universidad Nacional de Moquegua, Moquegua 18001, Peru; erick_16_13@hotmail.com

**Keywords:** TCATA, texture profile, consumers, healthy meat products

## Abstract

This study aimed to investigate the incorporation of micronized salt (MS) to reduce sodium content in fresh sausages while preserving technological, chemical, textural, and sensory characteristics. Four treatments were prepared: control (C) with 2.0% regular salt; M2.0% with 2.0% micronized salt; M1.5% with 1.5% micronized salt; and M1.0% with 1.0% micronized salt, containing 1004, 1133, 860, and 525 mg of sodium/100 g of product, respectively. To characterize the samples, analyses of sodium content, cooking loss, relative myoglobin content, and instrumental color were carried out. The sensory analysis was performed using the Temporal-Check-All-That-Apply (TCATA) method. Half of the micronized salt treatment was mixed with the fat during the processing of the fresh sausages. It was possible to achieve a 50% reduction in sodium (M1.0%) in the fresh sausages without negative effects on most technological, chemical, and textural parameters, which did not differ from the control treatment (C). Conversely, “chewiness” decreased in M2.0% compared to the control (C) due to mixing micronized salt with the fat. The sodium reduction did not impact the temporal sensory profile and overall liking. Therefore, using micronized salt in fresh sausages reduces sodium content without affecting sensory traits and product stability.

## 1. Introduction

In recent decades, there has been a significant increase in sudden deaths caused by non-communicable chronic diseases, primarily cardiovascular issues and arterial hypertension [1]. In this context, excessive sodium consumption is considered a contributing factor [2]. As a result, reducing sodium intake has become a global concern.

The majority of sodium intake in the population is in the form of sodium chloride (NaCl) and comes from food products, such as processed meat, representing about 20 to 30% of daily intake [3]. Fresh sausage is a popular processed meat product in Brazil, with coarsely ground pork (minimum 12%) and pork fat (maximum 30%) mixed with other ingredients (salt, additives, spices, and condiments) and embedded in natural or artificial casings [4]. However, its high NaCl content makes it a significant source of sodium in the human diet. Consequently, seeking technological strategies to promote sodium reduction in fresh sausages without altering the original traits is essential and challenging because sodium chloride affects fresh sausage texture and flavor intensity based on protein solubilization, activation of protein extraction [5], enhancing hydration, and water holding capacity (WHC). Furthermore, experiments carried out in laboratories and pilot plants can only sometimes be scalable to large production [6].

Many scientific studies on meat products aim to reduce or replace sodium chloride (NaCl) with alternative ingredients like non-sodium (substitutes) salts or flavor enhancers, or by modifying crystal size [5,6,7,8,9,10,11]. Reducing the sodium content in meat products is made possible with the use of salt substitutes. These substitutes are ingredients that function similarly to salt, but with significantly less or no sodium in comparison to table salt (sodium chloride). Potassium chloride (KCl), magnesium chloride (MgCl_2_), calcium chloride (CaCl_2_), and inorganic blend salts [12] are some examples of salt substitutes. One of the most commonly used substitutes for NaCl is KCl due to its similar functionality and structure. In a study comparing the sensory perception of various salts in an aqueous solution to that of sodium chloride, KCl was the only one to present a similar temporal sensory profile [13]. Another study evaluating the partial replacement of NaCl in frankfurter sausages with KCl and CaCl_2_ found that CaCl_2_ caused low stability in the emulsion when used to replace 50% of the NaCl [14]. The samples with 50% NaCl replacement with KCl decreased saltiness and preserved the bitterness. In another work, treatments containing 70% NaCl, 20% KCl, 3.5% maltodextrin, 4% lysine, 1% alanine, 0.5% citric acid, and 1% calcium lactate had a less bitter taste than sausages made with 70% NaCl and 30% KCl. There was no statistical difference in the acceptability of the samples with 100% NaCl and the samples with the afore mentioned percentage composition [15].

Various strategies for reducing sodium in foods, such as non-sodium salt substitutes, alternative ingredients, and flavor enhancers, encounter regulatory hurdles in different countries. For example, in Brazil, potassium chloride has yet to be specifically authorized for use in meat products [16]. An effective way to reduce sodium content in sausages is using micronized salt, which has smaller particle sizes than regular NaCl. Research indicates that 70–95% of salt is not dissolved in saliva [17] and using smaller particle sizes can improve the perception of saltiness [8]. Studies have shown that beef burgers with only 1% micronized salt have similar sensory characteristics to those with 1.5% regular NaCl, indicating that reducing NaCl by 30% is possible without affecting the sensory properties [18,19].

In this scenario, industries can reformulate products, adjusting to the original formulation and aiming for products with lower sodium content. However, it is important to ensure that modifications align with the consumer’s sensory expectations. As Oliveira et al. [20] have observed, consumers often hesitate to compromise sensory quality for health reasons. It is also worth noting that reformulating a product can introduce complex sensory changes. Hence, relying solely on static sensory tests may not provide a complete perception, since they record only a picture. Studying the dynamic sensory profile of low-sodium fresh sausages seems imperative for comprehensively understanding the sensory phenomenon [21].

Dynamic sensory tests can provide more realistic insights than static tests as they capture responses often concealed during food consumption. The act of consuming a particular food involves multisensory experiences. Therefore, the choice of a sensory test should not be statically limited to a fixed set of sensory attributes. Instead, it should be dynamic and aim to obtain detailed consumer opinion results [22,23].

In this context, Temporal Check-All-That-Apply (TCATA) is a new dynamic method that aims to identify all sensory attributes perceived during tasting. TCATA has been shown to be effective in studying how sensory attributes of food products can be applied [24]. Additionally, these attributes have been linked to the overall liking, which allows for identifying drivers of liking, i.e., when attributes are present in the sensory description, they can modify the product’s liking. In this framework, this study examines the effect of micronized salt on the sensory, technological, chemical, and textural characteristics of fresh sausages.

## 2. Materials and Methods

The experiment was conducted at the Meat Quality and Processing Laboratory of Departamento de Agroindústria, Alimentos e Nutrição (LAN) of the Escola Superior de Agricultura “Luiz de Queiroz” (ESALQ), Universidade de São Paulo (USP).

### 2.1. Raw Materials and Ingredients

The pork meat was obtained from 5-month-old commercial breed animals (Agroceres Pig). The pork backfat was donated by a commercial slaughterhouse Frigodeliss Ltda (Capivari-SP). Meat and fat were transported in thermal boxes to the laboratory. They were kept frozen (−18 °C) until the production of fresh sausages. The micronized salt (MS) was obtained by sieving regular salt (RS) by manually sieving using a stainless-steel sieve with a 60-mesh size, resulting in a particle size of 168.86 ± 1.66 μm, yielding micronized salt (MS), according to Rios-Mera et al. [19]. The remaining ingredients were provided by Ibrac (Rio Claro, SP, Brazil).

### 2.2. Manufacture of Fresh Sausages

Four treatments were produced: control (C) with 2.0% regular salt, M2.0% with 2.0% micronized salt, M1.5% with 1.5% micronized salt, and M1.0% with 1.0% micronized salt, as shown in Table 1. The entire experiment was conducted in a Randomized Complete Block Design (RCBD) considering two independent processing batches as blocks (*n* = 40 fresh sausages/treatment/batch). In the manufacturing of the fresh sausages, pork meat (Semimembranosus) and pork backfat were ground using 8 mm and 6 mm grinding discs, respectively. For the treatments using micronized salt (M2.0%, M1.5%, and M1.0%), half of the MS was mixed with the fat, and the other half was combined with the meat batter and other ingredients. The mixture was homogenized for 2 min before being stuffed into natural pork casings (Canova, 32 mm caliber) soaked in a 1% saline solution. In the control treatment (C), regular salt was mixed with the meat and fat before homogenization. Subsequently, the fresh sausages were vacuum packed (−73 cm Hg, Selovac 300 B vacuum packer, Sao Paulo, Brazil) and stored at −18 °C for subsequent analyses.

### 2.3. Characterization of Fresh Sausages

#### 2.3.1. Moisture and Ash

We determined, in triplicate, the moisture and ash content of the fresh sausages according to AOAC guidelines [25], using the following equations:

%Moisture = [(Final weight − initial weight)/(Sample weight)] × 100

%Ash = [(Final ash weight − crucible weight)/(Sample weight)] × 100

#### 2.3.2. pH and aw

The pH values were determined in the cooked fresh sausages, with measurements taken in triplicate using a properly calibrated pH meter (buffer solutions 4.0, 7.0, and 10.0) equipped with a temperature sensor electrode (Lucadema model, LUCA-210, São José do Rio Preto, Brazil). The water activity (aw) was determined at 25 °C in raw fresh sausage samples using an AquaLab^®^ 4TEV water activity instrument (Decagon, Pullman, WA, USA).

#### 2.3.3. Instrumental Color

The MiniScan^®^XE Plus spectrophotometer (Hunter Associates Laboratory Inc., Reston, VA, USA) was used to determine the lightness (L*), redness (a*), yellowness (b*), chroma (C*), and hue angle (H*) of raw fresh sausages exposed on Petri dishes. Before taking color measurements, the equipment underwent calibration using a white ceramic tile set to Y = 93.7, x = 0.3160 and y = 0.3323. The measurement area was 8 mm in diameter, the viewing angle was 10 degrees, and A10 illuminant was used. Each sample was measured in triplicate. To determine the values of chroma (C*) and hue angle (H*), we employ the following equations:

Chroma (C*, saturation index = [(a*^2^ + b*^2^)^0.5^]);

hue angle (Hue = [tan inverse (b*/a*)]);

#### 2.3.4. Relative Myoglobin Content

The relative content of myoglobin (oxymyoglobin OMb and metmyoglobin MMb) was determined according to AMSA [26]. Raw fresh sausage samples were exposed on Petri dishes and using a calibrated MiniScan^®^XE Plus portable spectrophotometer (Hunter Associates Laboratory Inc., Reston, VA, USA), reflectance spectra were obtained from 400 to 700 nm in 10 nm increments. Linear interpolation was used to calculate the reflectance at 473, 525, and 572 nm, subsequently converted into absorbance (A = log (1/R)). The absorbance values were used in equations provided by [18] to quantify the relative myoglobin content. The absorbance values were used in equations provided by AMSA [26] to quantify the relative myoglobin content:

%MMb = (1.395 − (A572 − A730)/ (A525 − A730)) × 100

%DMb = [2.375 × (1 − (A473 − A730)/ (A525 − A730))] × 100

%OMb = 100 − (%MMb + %DMb)

#### 2.3.5. Cooking Loss

Cooking loss was determined following the methodology described by Honikel [27] and calculated according to the equation:

%Cooking loss = [(Raw weight − cooked weight)/ (Raw weight)] × 100

#### 2.3.6. Instrumental Texture

The textural properties of the fresh sausages were analyzed through Texture Profile Analysis (TPA) following the recommendations of Bourne [28] using a TA-XT texture analyzer (Stable Micro Systems, Godalming, UK). For each treatment, six cylinders were sampled using a stainless-steel cutter with a height of 2 cm. Then, cooked samples were compressed twice axially to 50% of their original height at a pre-test speed of 1 mm/s and 5 mm/s, using a P/35 probe (Stable Micro Systems, Godalming, UK). The parameters considered were hardness (N), springiness (mm), cohesiveness (dimensionless), and chewiness (N).

#### 2.3.7. Sodium Content

The quantification of sodium content in sausage samples was performed following the methodology described by Paez et al. [25] and Almeida et al. [29]. Initially, 5 g of samples were incinerated at 550 °C (Jung, Blumenau, Santa Catarina, Brazil). The resulting ashes were cooled to room temperature and then solubilized in 15 mL of 25% nitric acid solution (250 mL of nitric acid in 750 mL of distilled water). The samples were analyzed in triplicate using a flame photometer (Digimed, model DM63, São Paulo, Brazil). For calculation, a blank sample was used as a control.

#### 2.3.8. Microbiological Analysis

Before conducting sensory analysis of fresh sausages, the microbiological quality of samples was assessed considering the examination of *Escherichia coli*, mesophilic aerobic bacteria, and *Salmonella* spp. following the American Public Health Association [30].

#### 2.3.9. Sensory Analysis

##### Consumers

For the sensory analysis, we recruited 109 regular consumers of fresh pork sausages from the campus community, including students, staff, and visitors, all over 18 years old. Initially, the participants read and signed the Informed Consent Form, previously approved by the Ethics Committee for Human Research at ESALQ/USP (protocol number 6.087.327).

##### Procedure

The sensory tests were conducted at the Sensory Laboratory of Departamento de Agroindútria, Alimentos e Nutrição (LAN) at ESALQ/USP. The samples (~10 g) were served monadically to consumers with three-digit codes following the Williams Latin Square design. Consumers performed the TCATA test, followed by the overall liking test. Water and unsalted crackers were provided to cleanse the palate between the samples. The data were collected in the Compusense Cloud software (Compusense Inc., Guelph, ON, Canada) via tablets with the Android operating system.

##### Temporal-Check-All-That-Apply

The TCATA test followed previous research protocols [10,31]. The participants were asked to select sensory attributes from a predetermined list (salty, juicy, firm, off-flavor, dry, and tender) that describe each sample over time. They could check as many attributes as they felt were necessary while tasting.

##### Overall Liking

Immediately after the TCATA test, consumers were instructed to rate their overall liking using a 9-point hedonic scale ranging from 1 = extremely dislike to 9 = extremely like.

### 2.4. Data Analysis

The experiment was implemented in an RCBD, considering each processing as a block. The instrumental results (in triplicate) underwent a mixed analysis of variance (ANOVA), with treatment as a fixed factor and processing as a random factor. When treatment was significant (*p* < 0.05), the Tukey test was applied for pairwise comparison.

The proportions of citations for attributes and products over time were represented in curve plots. Thick solid lines represent significant reference curves for sensory attributes [32]. The overall liking of pork sausage was submitted to ANOVA considering treatments, consumers, and presentation order as factors. When applicable, the Tukey test was employed to explore the differences among treatments. All inferential tests were conducted at 5% of significance. 

## 3. Results and Discussion

### 3.1. Physicochemical Analysis, Yield Properties, Texture Profile, and Sodium Content

The decrease in sodium levels in the formulations did not affect the moisture, pH, aw, cooking loss, and springiness values, as shown in Table 2. These results indicate that NaCl is effective in retaining components such as water at up to 1.0% NaCl in the sausage formulation. Similar results were observed by Saavedra et al. [33] in fish burgers, at NaCl reduction levels of 1.0% to 0.5%. Furthermore, the moisture content remained within the guidelines of Brazilian regulations for fresh sausages, which stipulate that values should not exceed 70% [34]. This information is important for the industrial application of the product.

It was observed that the sodium content in sample M2.0% was not significantly different from the control sample. This was expected because both samples contained the same amount of added salt, although the salt used in M2.0% was micronized; that is, the particle size of the NaCl did not affect the quantification of ash and sodium when both RS and MS were added to the same concentration of the sausages. On the other hand, since NaCl is a mineral that constitutes ashes, it is expected that ashes decrease with lower NaCl content, as observed in the 1.5% and 1.0% NaCl treatments. The same trend was observed for sodium content.

The treatment with 1% NaCl (M1.0%) resulted in a significant reduction of 52.3% in sodium levels compared to the control. This makes it a promising candidate to obtain the “low sodium” label as per Regulation (EC) nº 1924/2006 [35]. This outcome is particularly beneficial for consumers of fresh sausages as it can help reduce their exposure to high sodium levels, lowering the risk of developing cardiovascular diseases, as suggested by Ruusunen et al. [5].

Regarding TPA, the springiness of the burgers was not affected by the decrease in sodium content. However, the samples’ hardness, cohesiveness, and chewiness showed a significant reduction (as shown in Table 2). The importance of salt in meat products has been extensively discussed in the literature. Sodium chloride (NaCl) dissolves myofibril proteins in the meat matrix, which enhances the bonding capacity between water and fat, resulting in a firmer texture profile [36]. The salt content reduction in the formulations may have led to decreased extraction of myofibril proteins, resulting in reduced hardness, cohesiveness, and chewiness. These findings are consistent with a previous study that observed similar results in fresh sausages with reduced sodium content [37]. On the other hand, when comparing sausages with equal salt content (treatments C and M2.0%), it is possible to observe that using micronized salt leads to a decrease of eight units in chewiness. This decrease may be attributed to the mixture of micronized salt with fat, which could hinder the interaction between salt and myofibrillar proteins and ultimately affect the texture of the sausage, as previously explained.

### 3.2. Instrumental Color and Relative Myoglobin Content

Table 3 displays the instrumental color characteristics such as L*, a*, b*, C*, and H* and the relative myoglobin content of fresh sausages with different concentrations of micronized NaCl. Replacing regular salt (RS) with micronized salt (MS) and reducing salt content did not affect the color responses a*, b*, C*, and H*. These findings are similar to those of a study conducted by Honikel et al. [27] that examined the reduction of encapsulated salt in carnauba wax in fresh sausages over 90 days of vacuum packaging and refrigerated storage.

The study found no differences in instrumental color, indicating that reducing sodium or using non-sodium salt in meat products does not hurt the final product’s color [38,39]. Conversely, the M1.0% treatment showed a lower L* value and a higher percentage of metmyoglobin, as shown in Table 3, when compared to the other treatments (*p* < 0.05). The lower L* values observed in this treatment are related to a higher concentration of metmyoglobin. This change imparts a brown color to the meat product, reducing L* values [40]. The decrease in L* values could be associated with the oxidative process of lipids and proteins responsible for color deterioration in meat products [41]. In addition, sodium reduction is responsible for altering the water retention capacity, impairing color development due to changes in light dispersion by the meat matrix [5].

### 3.3. Microbiological Characterization

The microbiological quality of the fresh sausages formulated in this paper is safe for human consumption according to the Brazilian regulations RDC No. 724, dated 1 July 2022, and IN No. 161, dated 1 July 2022 [16]. The samples exhibited average values of <3.0 MPN for *Escherichia coli*, mean counts of mesophilic aerobic bacteria at 2.4 × 1 10^3^ CFU/g, and the absence of *Salmonella* spp. in 25 g of sample.

### 3.4. Temporal Sensory Profile and Overall Liking

**Figure 1:**
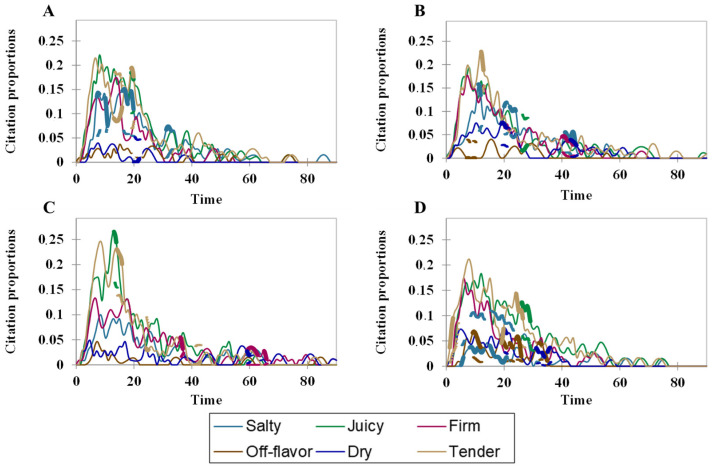
Citation proportions of sensory attributes for fresh sausages. (**A**) = C (control formulation), (**B**) = M2.0% (2.0% micronized salt), (**C**) = M1.5% (1.5% micronized salt), (**D**) = M1.0% (1.0% micronized salt).

Figure 1 displays the TCATA citation curves for the fresh sausage samples. All treatments exhibited a similar pattern in the initial 20 s of tasting. Attributes such as juiciness, tenderness, and firmness received the highest citation proportions. However, differences among the treatments became apparent when assessing the line thickness for each attribute.

According to Castura et al. [32], an increase in line thickness indicates that a specific attribute is more cited in one treatment than others. Treatments C and M2.0% exhibit the highest proportions of salty flavor, as they contain the most NaCl. However, it is noteworthy that micronized salt (M2.0%) increased tenderness compared to regular salt (C). This phenomenon can be attributed to the improved penetration of salt into the meat matrix and an increase in the extraction of myofibrillar proteins. Similarly, Aheto et al. [42] observed great NaCl penetration in dry-cured pork when using NaCl crystals of 0.55 mm.

In treatment M1.0%, the salty attribute had the lowest proportions. However, the other attributes exhibited behavior similar to samples C and M2.0%, indicating that the reduction in salty taste did not significantly impact the consumers’ perceptions of the other attributes. Among all the treatments, treatment M1.5% stood out, showing the highest citations for juiciness and tenderness.

As mentioned by Rios-Mera et al. [10], reducing NaCl in beef burgers increased the proportions of negative attributes. However, in contrast to this scenario, our study reduced 50% of NaCl without a high proportion of negative sensory attributes. These results suggest that the use of micronized salt may be more effective in fresh sausages than in burgers.

**Figure 2:**
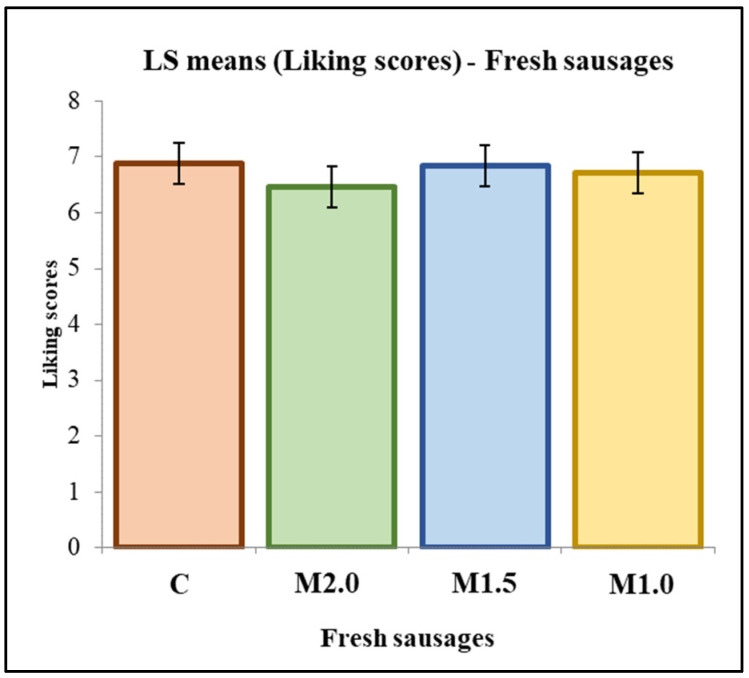
Overall liking of fresh sausages. C = control formulation, M2.0% = 2.0% micronized salt, M1.5% = 1.5% micronized salt, M1.0% = 1.0% micronized salt.

Figure 2 illustrates the overall liking scores, revealing no statistically significant differences among the treatments. The assessments conducted on a 9-point hedonic scale converged to values close to 7 (like moderately). Even with a 50% reduction in sodium content in the fresh sausages, consumers could not detect a difference compared to the control treatment (C). Micronized salt appears to have played a significant role in this outcome, intensifying the salty flavor in the low-sodium treatments.

The technique of using micronized salt represents a simple and easily applicable approach in the food industry, utilizing an ingredient (NaCl) already present in the original formulations. This technique involves the reduction of the size of salt particles, a process that can be carried out using accessible equipment, such as a sieve, for example. In light of this, the results obtained in this study are of utmost importance, highlighting the feasibility of developing healthier products. These products can meet the demands for foods with lower sodium content without compromising sensory acceptance and textural, technological, and color characteristics perceived by consumers.

## 4. Conclusions

The use of micronized salt (MS) enclosed within the fat layer has enabled a reduction of sodium content of up to 50% in fresh sausages (M1.0%) without any significant changes in technological, chemical, textural, and microbiological characteristics. When MS was combined with fat, the chewiness of the product decreased in comparison to the control treatment at the same concentration. As far as the sensory aspect is concerned, the TCATA attribute mentions were similar for all the treatments, and consumers did not notice any differences in overall liking. Additional research is needed to assess the oxidative behavior of proteins and lipids in fresh sausages with reduced sodium content through micronization.

## Figures and Tables

**Table 1:** Formulations of fresh sausages.

Component (%)	Treatments			
	C	M2.0%	M1.5%	M1.0%
Pork meat	70.88	70.88	71.38	71.88
Pork backfat	20	20	20	20
Water	6	6	6	6
Regular salt	2	–	–	–
Micronized salt	–	2	1.5	1.0
White ground pepper	0.1	0.1	0.1	0.1
Garlic powder	0.2	0.2	0.2	0.2
Sodium nitrite	0.02	0.02	0.02	0.02
Sodium erythorbate	0.3	0.3	0.3	0.3
Monosodium glutamate	0.3	0.3	0.3	0.3

C = control formulation, M2.0% = 2.0% micronized salt, M1.5% = 1.5% micronized salt, M1.0% = 1.0% micronized salt.

**Table 2:** Physicochemical analysis, sodium, yield, and texture properties of fresh sausages.

Characteristics	Treatments					
	C	M2.0%	M1.5%	M1.0%	SEM	*p*-Value
Moisture (%)	40.62	41.66	40.68	40.70	0.383	0.236
Ash (%)	3.57 ^a^	3.78 ^a^	2.85 ^b^	2.72 ^b^	0.104	<0.0001
pH	6.12	6.18	6.18	6.23	0.033	0.184
aw	0.966	0.964	0.968	0.967	0.002	0.710
Sodium (mg/100 g)	1004 ^a^	1133 ^a^	860 ^b^	525 ^c^	0.061	<0.0001
Cooking loss (%)	24.58	22.48	20.04	24.27	1.785	0.053
Hardness (N)	108.43 ^a^	103.65 ^a^	89.86 ^b^	83.96 ^b^	1.890	<0.0001
Springiness	0.906	0.886	0.908	0.899	0.006	0.171
Cohesiveness	0.693 ^a^	0.656 ^ab^	0.666 ^ab^	0.612 ^b^	0.020	0.023
Chewiness (N)	68.10 ^a^	60.34 ^b^	54.35 ^c^	46.04 ^d^	1.573	<0.0001

Different letters between mean values at the same row mean statistical differences among treatments (*p* < 0.05). C = control formulation, M2.0% = 2.0% micronized salt, M1.5% = 1.5% micronized salt, M1.0% = 1.0% micronized salt. SEM: Standard error of the mean.

**Table 3:** Instrumental color of fresh sausages.

Characteristics of Raw Sausages	Treatments					
	C	M2.0%	M1.5%	M1.0%	SEM	*p*-Value
Lightness (L*)	62.17 ^ab^	63.91 ^a^	62.65 ^ab^	61.82 ^b^	0.602	0.019
Redness (a*)	19.04	17.38	18.15	17.46	0.722	0.231
Yellowness (b*)	20.77	20.01	20.62	20.23	0.371	0.424
Chroma (C*)	28.19	26.51	27.47	26.74	0.748	0.295
Hue (H*)	47.63	49.06	48.65	49.29	0.674	0.163
Metmyoglobin (%)	31.64 ^b^	36.59 ^ab^	36.86 ^ab^	37.89 ^a^	1.422	0.018
Oxymyoglobin (%)	76.45	71.40	71.99	70.31	1.661	0.092

Different letters between mean values at the same row means statistical differences (*p* < 0.05). C = control formulation, M2.0% = 2.0% micronized salt, M1.5% = 1.5% micronized salt, M1.0% = 1.0% micronized salt. SEM: Standard error of the mean.

## Data Availability

Data are contained within the article.

## References

[1] Grillo A., Salvi L., Coruzzi P., Salvi P., Parati G. (2019). Sodium Intake and Hypertension. Nutrients.

[2] He F.J., Tan M., Ma Y., MacGregor G.A. (2020). Salt Reduction to Prevent Hypertension and cardiovascular disease. J. Am. Coll. Cardiol..

[3] Gullón P., Astray G., Gullón B., Franco D., Campagnol P.C.B., Lorenzo J.M. (2021). Inclusion of seaweeds as healthy approach to formulate new low-salt meat products. Curr. Opin. Food Sci..

[4] ABIPECS (2012). SUÍNA-ABIPECS, A.C. Relatório Annual. https://abcs.org.br/.

[5] Ruusunen M., Puolanne E. (2005). Reducing sodium intake from meat products. Meat Sci..

[6] do Nacimento R., Vidal V.A., de Souza Paglarini C., Munekata P.E., Lorenzo J.M., Barros J.C., Cristianini M., Pollonio M.A. (2024). Effect of processing (cutter versus continuous emulsifier) and sodium reduction on the physicochemical properties of bologna sausages. J. Sci. Food Agric..

[7] Cruz-Romero M.C., O’Flynn C.C., Troy D., Mullen A.M., Kerry J.P. (2021). The Use of Potassium Chloride and Tapioca Starch to Enhance the Flavour and Texture of Phosphate- and Sodium-Reduced Low Fat Breakfast Sausages Manufactured Using High Pressure-Treated Meat. Foods.

[8] da Rosa J.L., Rios-Mera J.D., Castillo C.J., Lorenzo J.M., Pinton M.B., Dos Santos B.A., Correa L.P., Henn A.S., Cichoski A.J., Flores E.M. (2023). High-power ultrasound, micronized salt, and low KCl level: An effective strategy to reduce the NaCl content of Bologna-type sausages by 50%. Meat Sci..

[9] Inguglia E.S., Zhang Z., Tiwari B.K., Kerry J.P., Burgess C.M. (2017). Salt reduction strategies in processed meat products—A review. Trends Food Sci. Technol..

[10] Rios-Mera J.D., Saldaña E., Cruzado-Bravo M.L., Martins M.M., Patinho I., Selani M.M., Valentin D., Contreras-Castillo C.J. (2020). Impact of the content and size of NaCl on dynamic sensory profile and instrumental texture of beef burgers. Meat Sci..

[11] Selani M.M., Ramos P.H., Patinho I., França F., dos Santos Harada-Padermo S., Contreras-Castillo C.J., Saldaña E. (2022). Consumer’s perception and expected liking of labels of burgers with sodium reduction and addition of mushroom flavor enhancer. Meat Sci..

[12] Tan H., Tan T., Easa A.M. (2022). The use of salt substitutes to replace sodium chloride in food products: A review. Int. J. Food Sci. Technol..

[13] Feltrin A.C., de Souza V.R., Saraiva C.G., Nunes C.A., Pinheiro A.C.M. (2015). Sensory study of different sodium chloride substitutes in aqueous solution. Int. J. Food Sci. Technol..

[14] Horita C.N., Messias V.C., Morgano M.A., Hayakawa F.M., Pollonio M.A.R. (2014). Textural, microstructural and sensory properties of reduced sodium frankfurter sausages containing mechanically deboned poultry meat and blends of chloride salts. Food Res. Int..

[15] Chen J., Hu Y., Wen R., Liu Q., Chen Q., Kong B. (2019). Effect of NaCl substitutes on the physical, microbial and sensory characteristics of Harbin dry sausage. Meat Sci..

[16] Brazil Agência Nacional de Vigilância Sanitária. Resolução de Diretoria Colegiada No. 272, de 14 de Março de 2019. https://www.cidasc.sc.gov.br/inspecao/files/2019/03/RESOLUC%CC%A7A%CC%83O-DA-DIRETORIA-COLEGIADA-RDC-N%C2%BA-272-DE-14-DE-MARC%CC%A7O-DE-2019-Dia%CC%81rio-Oficial-da-Unia%CC%83o-Imprensa-Nacional.pdf.

[17] Quilaqueo M., Duizer L., Aguilera J.M. (2015). The morphology of salt crystals affects the perception of saltiness. Food Res. Int..

[18] Rios-Mera J.D., Saldaña E., Patinho I., Selani M.M., Contreras-Castillo C.J. (2021). Enrichment of NaCl-reduced burger with long-chain polyunsaturated fatty acids: Effects on physicochemical, technological, nutritional, and sensory characteristics. Meat Sci..

[19] Rios-Mera J.D., Saldaña E., Cruzado-Bravo M.L., Patinho I., Selani M.M., Valentin D., Contreras-Castillo C.J. (2019). Reducing the sodium content without modifying the quality of beef burgers by adding micronized salt. Food Res. Int..

[20] Oliveira D., Antúnez L., Giménez A., Castura J.C., Deliza R., Ares G. (2015). Sugar reduction in probiotic chocolate-flavored milk: Impact on dynamic sensory profile and liking. Food Res. Int..

[21] Visalli M., Galmarini M.V., Schlich P. (2023). Use of temporal sensory evaluation methods with consumers: A position paper. Curr. Opin. Food Sci..

[22] de Souza Paglarini C., Vidal V.A., Dos Santos M., Coimbra L.O., Esmerino E.A., Cruz A.G., Pollonio M.A. (2020). Using dynamic sensory techniques to determine drivers of liking in sodium and fat-reduced Bologna sausage containing functional emulsion gels. Food Res. Int..

[23] Auriema B.E., Correa F.J., Silva R., Soares P.T., Lima A.L., Vidal V.A., Raices R.S., Pollonio M.A., Luchese R.H., Esmerino E.A. (2022). Fat replacement by green banana biomass: Impact on the technological, nutritional and dynamic sensory profiling of chicken mortadella. Food Res. Int..

[24] Ares G., Alcaire F., Antúnez L., Vidal L., Giménez A., Castura J.C. (2017). Identifica-tion of drivers of (dis)liking based on dynamic sensory profiles: Comparison of Temporal Dominance of Sensations and Temporal Check-all-that-apply. Food Res. Int..

[25] Paez V., Barrett W.B., Deng X., Diaz-Amigo C., Fiedler K., Fuerer C., Hostetler G.L., Johnson P., Joseph G., Konings E.J. (2016). AOAC SMPR® 2016.002. J. AOAC Int..

[26] AMSA (2012). AMSA Meat Color Measurement Guidelines.

[27] Honikel K.O. (1998). Reference methods for the assessment of physical characteristics of meat. Meat Sci..

[28] Bourne M.C. (1978). Texture profile analysis. Food Technol..

[29] Almeida M.A.D., Villanueva N.D.M., Pinto J.S.D.S., Saldaña E., Contreras-Castillo C.J. (2016). Sensory and physicochemical characteristics of low sodium salami. Sci. Agric..

[30] Salfinger Y., Tortorello M.L. (2015). Compendium of Methods for the Microbiological Examination of Foods.

[31] Ares G., Castura J.C., Antúnez L., Vidal L., Giménez A., Coste B., Picallo A., Beresford M.K., Chheang S.L., Jaeger S.R. (2016). Comparison of two TCATA variants for dynamic sensory characterization of food products. Food Qual. Prefer..

[32] Castura J.C., Baker A.K., Ross C.F. (2016). Using contrails and animated sequences to visualize uncertainty in dynamic sensory profiles obtained from temporal check-all-that-apply (TCATA) data. Food Qual. Prefer..

[33] Saavedra A.R., Rios-Mera J.D., Imán A., Vásquez J., Saldana E., Siche R., Tello F. (2022). A sequential approach to reduce sodium chloride in fresh-water fish burgers considering chemical, texture, and consumer sensory responses. LWT.

[34] Brazil (2000). Ministério da Agricultura, Pecuária e Abastecimento—MAPA. Instrução Normativa No 4. Anexo III—Regulamento Técnico de Identidade e Qualidade de Linguiça.

[35] Regulation EC No 1924/2006 of the European Parliament and of the Council of 20 December 2006 on nutrition and health claims made on food. Off. J. Eur. Union..

[36] Desmond E. (2006). Reducing salt: A challenge for the meat industry. Meat Sci..

[37] Beck P.H.B., Matiucci M.A., Neto A.A.M., Feihrmann A.C. (2021). Sodium chloride reduction in fresh sausages using salt encapsulated in carnauba wax. Meat Sci..

[38] Pires M.A., Munekata P.E., Baldin J.C., Rocha Y.J., Carvalho L.T., dos Santos I.R., Barros J.C., Trindade M.A. (2017). The effect of sodium reduction on the microstructure, texture and sensory acceptance of Bologna sausage. Food Struct..

[39] da Silva Araujo D.H., de Souza Rodrigues R.T., da Costa M.M., de Miranda J.O., de Lira-Alencar N.R., Queiroz M.A., de Alencar M.G., Oliveira T.P., Neto A.F., Bonfá H.C. (2021). Reduction of sodium content in frozen goat sausage using different types of salt. LWT.

[40] de Oliveira R.F., da Costa Henry F., do Valle F., de Oliveira D.B., do Santos Junior A.C., de Resende E.D., Junior J.D., Martins M.L. (2020). Effect of the fruit aqueous extract of Brazilian pepper tree (*Schinus terebinthifolius*, Raddi) on selected quality parameters of frozen fresh pork sausage. J. Agric. Food Res..

[41] Amaral A.B., Silva M.V.D., Lannes S.C.D.S. (2018). Lipid oxidation in meat: Mechanisms and protective factors—A review. Food Sci. Technol..

[42] Aheto J.H., Huang X., Xiaoyu T., Bonah E., Ren Y., Alenyorege E.A., Chunxia D. (2019). Investigation into crystal size effect on sodium chloride uptake and water activity of pork meat using hyperspectral imaging. J. Food Process. Preserv..

